# Fluorogenic Substrates for *In Situ* Monitoring of Caspase-3 Activity in Live Cells

**DOI:** 10.1371/journal.pone.0153209

**Published:** 2016-05-11

**Authors:** Ana M. Pérez-López, M. Lourdes Soria-Gila, Emma R. Marsden, Annamaria Lilienkampf, Mark Bradley

**Affiliations:** 1 School of Chemistry, EaStCHEM, University of Edinburgh, Joseph Black building, West Mains Road, Edinburgh EH9 3FJ, United Kingdom; 2 Department of Medicinal and Organic Chemistry, University of Granada, School of Pharmacy, Campus Cartuja s/n – 18071, Granada, Spain; The University of Texas MD Anderson Cancer Center, UNITED STATES

## Abstract

The *in situ* detection of caspase-3 activity has applications in the imaging and monitoring of multiple pathologies, notably cancer. A series of cell penetrating FRET-based fluorogenic substrates were designed and synthesised for the detection of caspase-3 in live cells. A variety of modifications of the classical caspase-3 and caspase-7 substrate sequence Asp-Glu-Val-Asp were carried out in order to increase caspase-3 affinity and eliminate caspase-7 cross-reactivity. To allow cellular uptake and good solubility, the substrates were conjugated to a cationic peptoid. The most selective fluorogenic substrate **27**, FAM-Ahx-Asp-Leu-Pro-Asp-Lys(MR)-Ahx, conjugated to the cell penetrating peptoid at the C-terminus, was able to detect and quantify caspase-3 activity in apoptotic cells without cross-reactivity by caspase-7.

## Introduction

Fluorogenic substrates and activity-based probes enable the study of protease function and have been used to elucidate the role of caspases in the progression of diseases such as cancer [1–6], neurodegenerative disorders [7–10], and sepsis [11,12]. Caspases are an important family of cysteine-dependent aspartate proteases that exist within cells as inactive zymogens with their cleavage giving active enzymes initiating cellular apoptosis [13–15]. Inappropriate control of this apoptotic machinery is implicated in many diseases [14,15], notably cancer [16,17]. As a part of the apoptotic cascade, executioner caspase-3 activates several important cellular substrates [14–20], such as PARP and ICAD, and its decreased activity is a prognostic indicator of chemoresistance in breast and ovarian cancer [21,22]. The ability to monitor caspase-3 activity *in situ* could provide a means, not only to elucidate its complex role in biological processes, but also to monitor the efficacy of anticancer drugs and to identify patients for whom discontinuation of ineffective toxic treatment is warranted, for example, due to acquired drug resistance [23].

Current methods are able to detect caspase-3 activity *in vitro* although they often display promiscuity and cannot be used to monitor caspase-3 within cells [24–26]. The majority of fluorogenic caspase-3 substrates are based on a four-residue recognition sequence Asp-Glu-Val-Asp (DEVD) [23,27,28], established via combinatorial library methods [29,30]; however, this sequence is also efficiently cleaved by caspase-7, which shares very similar substrate specificities with caspase-3. In mouse macrophages, 46 out of the 55 identified protein cleavage sites (within 48 proteins) were cleaved by both enzymes with only 3 sites specifically cleaved by caspase-3 [31]. Incorporation of unnatural amino acids into the recognition sequence has yielded caspase-3 substrates with increased selectivity [32,33]. Recently, Wolan achieved live cell imaging of caspase-3 activity in apoptotic cells, with selectivity over caspase-7, with a near-infrared fluorogenic pentapeptide substrate (incorporating the unnatural amino acid β-homo-Leu) coupled to a cell penetrating peptide derived from the viral SV40 Large T-antigen nuclear localising signal [34].

Here, FRET-based fluorogenic substrates, incorporating a tetrapeptide recognition sequence Asp-X_3_-X_2_-Asp, were designed and synthesised for the selective, *in situ* monitoring of caspase-3 activity. To allow detection in live cells, the substrates were conjugated to a cationic peptoid-based cellular delivery vehicle.

## Results and Discussion

### Substrate design and synthesis

**Fig 1:**
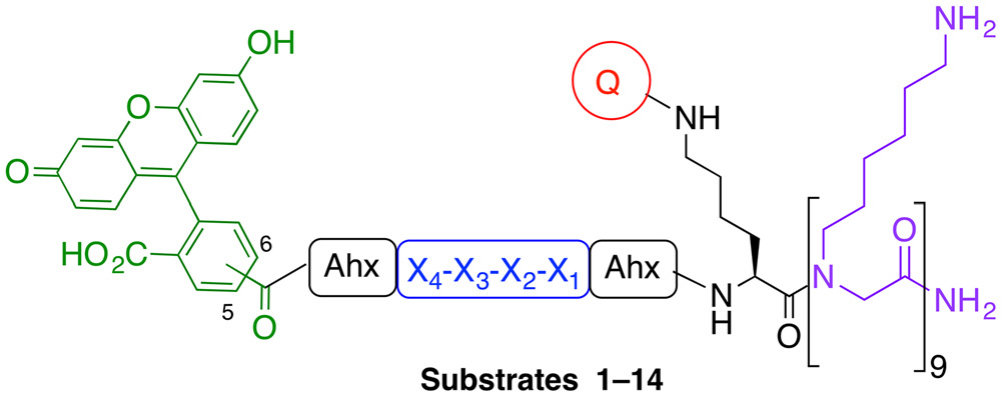
The design of the FRET-based fluorogenic tetrapeptide substrates for caspase-3 detection in live cells. The substrates bear 5(6)-carboxyfluorescein (λ_Ex/Em_ 488/528 nm) at the amino-terminus and a quencher coupled via a Lys side-chain. The recognition sequence is a tetrapeptide (Asp-X_3_-X_2_-Asp) with two variable positions (see Table 1). The C-terminus bears a “lysine-like” nonaresidue peptoid to enable cellular uptake.

In order to improve selectivity towards caspase-3 over caspase-7, permutations of the classical tetrapeptide substrate Asp-Glu-Val-Asp (X_4_-X_3_-X_2_-X_1_) were explored. All known caspase-3 substrates contain Asp at position X_1_ and 80% contain an Asp at the X_4_ position of the sequence. The positions X_3_ and X_2_ are more varied, with no clear amino acid preference being reported for the X_3_ position (~20% of the substrates contain Glu and ~15% Phe or Val at this position). Approximately 40% of known caspase-3 substrates contain Val at the X_2_ position; however, Pro at the X_2_ position is known to increase specificity for caspase-3 over caspase-7 [35]. With the aim of improving caspase-3 selectivity, X_3_ and X_2_ modifications of the tetrapeptide sequence were carried out, retaining Asp at X_1_ and X_4_ positions. The X_3_ position was changed to Pro, Gly, Ala, Leu, Asn and Val, and the X_2_ position had Val (substrates **1**–**9**) or Pro (substrates **10**–**14**) (Table 1) [36,37]. For each substrate, the corresponding d-amino acid sequence was synthesised as a control (compounds **15**–**24**, respectively, see ESI). 5(6)-Carboxyfluorescein was coupled to the N-terminus of the substrates via a 6-aminohexanoic acid (Ahx) spacer, and a quencher moiety was introduced next to the caspase cleavage site via Lys side chain modification (separated by an Ahx spacer from the Asp-X_3_-X_2_-Asp) (Fig 1). As the choice of the quencher can affect the rate of cleavage and level of background fluorescence, three different quenchers, methyl red (MR), Black Hole Quencher^®^-1 (BHQ1), and 5(6)-carboxytetraethylrhodamine (TAMRA) were evaluated. A cationic, “lysine-like” nonaresidue peptoid was incorporated onto the C-terminus to ensure cellular uptake of the substrates [38]. Unlike many common cell penetrating peptides [39–41] this peptoid is resistant to proteolysis, non-toxic *in vivo*, and has demonstrated a highly efficient cell entry profile [42–44]. In addition, peptoid-based delivery systems are not prone producing immunogenic responses associated with virus-derived sequences [45,46].

**Table 1:** The recognition sequence is a tetrapeptide (X_4_-X_3_-X_2_-X_1_) with two variable positions. X_1_ and X_4_ was Asp in all peptides. Three different quenchers, methyl red (MR), Black Hole Quencher^®^-1 (BHQ1), and 5(6)-carboxytetraethylrhodamine (TAMRA) were evaluated. As controls, substrates were also synthesised with the corresponding d-amino acid sequence (compounds **15**–**24**, respectively, see supporting information). For full structures, see Fig 1.

substrate	X_4_-X_3_-X_2_-X_1_	quencher (Q)
**1**	Asp-Glu-Val-Asp	MR
**2**	Asp-Glu-Val-Asp	TAMRA
**3**	Asp-Glu-Val-Asp	BHQ1
**4**	Asp-Pro-Val-Asp	MR
**5**	Asp-Gly-Val-Asp	MR
**6**	Asp-Ala-Val-Asp	MR
**7**	Asp-Leu-Val-Asp	MR
**8**	Asp-Asn-Val-Asp	MR
**9**	Asp-Val-Val-Asp	MR
**10**	Asp-Gly-Pro-Asp	MR
**11**	Asp-Ala-Pro-Asp	MR
**12**	Asp-Leu-Pro-Asp	MR
**13**	Asp-Asn-Pro-Asp	MR
**14**	Asp-Val-Pro-Asp	MR

The peptides **1–14** and controls **15**–**24** were synthesised on a Rink amide-functionalised aminomethyl polystyrene resin (1% DVB, 100–200 mesh, loading 1.2 mmol/g) using an Fmoc/^*t*^Bu-based strategy with microwave heating (S1 Fig) [47]. First, the nonapeptoid was synthesised using *N*-Fmoc-(6-Boc-aminohexyl)glycine [48] and DIC and Oxyma. Fmoc-Lys(Dde)-OH was coupled onto the peptoid, followed by Fmoc-Ahx-OH, and the substrate sequence (Asp-X_2_-X_1_-Asp), Fmoc-Ahx-OH, and 5(6)-carboxyfluorescein. The Lys side chain Dde protecting group was selectively removed with 2% hydrazine (v/v), followed by coupling of the carboxy-functionalised quencher. After deprotection and cleavage from the resin with TFA–TIS–DCM, peptides **1**–**24** were purified by preparative HPLC and analysed by MALDI-TOF MS.

### The effect of the quencher on caspase-3 cleavage

**Fig 2:**
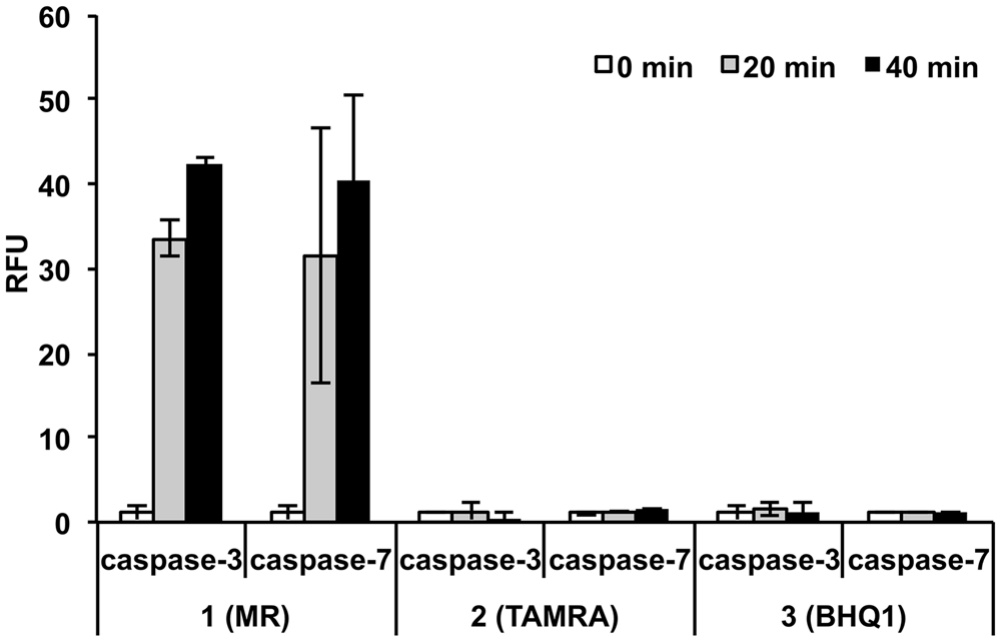
Relative increase in fluorescence intensity of the FRET-based peptides, bearing different quenchers (MR, TAMRA or BHQ1) on the Lys side chain, after incubation with caspase-3 and 7. The FRET-based peptides **1**, **2** and **3** (6 μM) were incubated with caspase-3 and 7 (20 nM) and fluorescence recorded at 10, 20 and 40 min (n = 3, normalised to zero).

To optimise the fluorogenic substrates, MR (λ_Abs_ 480 nm), TAMRA (λ_Abs_ 555 nm), and BHQ1 (λ_Abs_ 534 nm) were evaluated as quenchers for 5(6)-carboxyfluorescein (λ_Ex/Em_ 488/528 nm) in compounds **1**, **2** and **3**, using the classical substrate Asp-Glu-Val-Asp. Only **1** (MR as quencher) showed notable time-dependent increase in fluorescence upon incubation with caspase-3 and 7 (**2** and **3**, incorporating TAMRA and BHQ1, did not demonstrate significant increase in fluorescence) (Fig 2).

### Kinetic studies with Caspase-3 and 7

The ability of **1** and **5**–**24** (bearing MR as the quencher of choice) to act as s substrate for caspase-3, as well as caspase-7, was investigated by determining the catalytic efficiency (k_cat_/K_M_) for each substrate with both enzymes (Table 2). None of the d-amino acids containing sequences **15**–**24** or **4**, which has a Pro residue at position X_3_, showed any change in fluorescence intensity over time. With caspase-3, all the substrates with Val in X_2_ position (substrates **5**–**9**) showed similar catalytic efficiency (k_cat_/K_M_ 0.7–2.0 μM^-1^min^-1^) as Asp-Glu-Val-Asp (substrate **1**, 1.4 μM^-1^min^-1^). Substrate **5** (Asp-Gly-Val-Asp) showed 5-fold selectivity over caspase-7 (Table 2). Pro at the X_2_ position increased specificity for caspase-3 over caspase-7 [49], particularly with substrates **11** (Asp-Ala-Pro-Asp), **12** (Asp-Leu-Pro-Asp) and **14** (Asp-Val-Pro-Asp), which exhibited 8–20-fold selectivity over caspase-7, along with increased caspase-3 affinity (K_M_ 0.2–0.4 μM) and catalytic effiency (k_cat_/K_M_ 3.4–8.1 μM^-1^min^-1^).

**Table 2:** Kinetic analysis of the fluorogenic substrates. The kinetic parameters (n = 3) were determined for the fluorogenic substrates **1** and **5**–**14** (measured using substrate range of 0.1–8 μM) with caspase-3 and caspase-7. Substrate **4** or the d-amino acids containing **15**–**24** were not cleaved by either enzyme.

Caspase-3				Caspase-7		
	K_M_ (μM)	*Kcat* (min^-1^)	*kcat*/ K_M_ (μM^-1^min^-1^)	K_M_ (μM)	*kcat* (min^-1^)	*kcat*/ K_M_ (μM^-1^min^-1^)
**1**	0.6 ± 0.1	0.9 ± 0.04	1.4	0.5 ± 0.1	1.1 ± 0.1	2.1
**5**	0.3 ± 0.1	0.5 ± 0.03	1.7	1.2 ± 0.2	0.4 ± 0.1	0.3
**6**	1.5 ± 0.4	1.1 ± 0.1	0.7	14.0 ± 3.4	19.2 ± 1.6	1.4
**7**	0.8 ± 0.2	0.9 ± 0.1	1.2	0.9 ± 0.2	1.0 ± 0.1	1.1
**8**	0.4 ± 0.1	0.8 ± 0.04	2.0	0.6 ± 0.1	0.8 ± 0.03	1.3
**9**	1.8 ± 0.4	1.4 ± 0.1	0.8	4.1 ± 0.6	1.8 ± 0.1	0.4
**10**	0.7 ± 0.4	0.55 ± 0.2	0.8	0.3 ± 0.05	0.2 ± 0.01	0.6
**11**	0.4 ± 0.1	1.3 ± 0.1	3.4	4.9 ± 2.1	1.6 ± 0.3	0.3
**12**	0.2 ± 0.1	1.4 ± 0.2	5.8	1.4 ± 0.3	0.9 ± 0.07	0.7
**13**	0.8 ± 0.2	1.0 ± 0.2	1.2	1.2 ± 0.2	1.0 ± 0.06	0.8
**14**	0.2 ± 0.04	1.7 ± 0.1	8.1	3.6 ± 0.9	1.4 ± 0.2	0.4

To confirm how specific **11**, **12** and **14** were for caspase-3, these substrates were incubated with high enzyme concentrations (10–20 μM substrate, 0.4 μM enzyme) and the caspase-mediated cleavage analysed by MALDI-TOF MS. As expected, all the substrates were cleaved by caspase-3 at the X_1_ position (between Asp and the Ahx spacer), with the parent compound no longer detected after 2h; however, these substrates were also (partially) cleaved by caspase-7. Remarkably, MS analysis revealed that that caspase-7 exhibited a different cleavage pattern cleaving the substrates between the Ahx spacer and the Lys(MR) (S2–S4 Figs).

### Substrate optimisation

**Fig 3:**
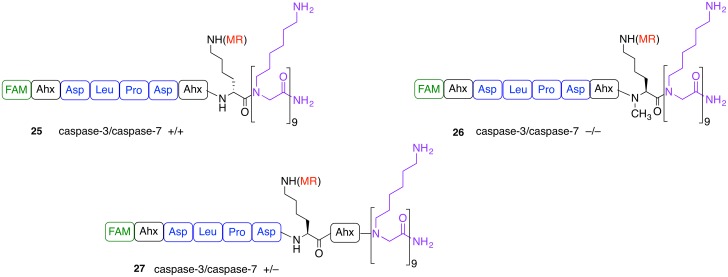
Structural modifications to the fluorogenic substrates with the aim of eliminating caspase-7 cross-reactivity. Substrate **25** has a D-Lys residue, **26** an *N*-Methyl-Lys, and in substrate **27** the Ahx spacer has been moved between the Lys and the peptoid moiety. Caspase-3 selectivity was achieved with **27**.

To eliminate the caspase-7 cross reactivity, three new substrates, all bearing Asp-Leu-Pro-Asp, were synthesised using d-Lys or *N*-Methyl-Lys as the quencher attachment point (**25** and **26**, respectively) and switching the position of the Ahx spacer (**27**) (Fig 3). Substrate **25** was cleaved by both caspase-3 and 7, whereas the *N*-methylated substrate **26** was not cleavage by either enzyme (S6 and S7 Figs). Substrate **27** showed good affinity for caspase-3 (K_M_ 1.1 ± 0.3 μM) with k_cat_ of 2.1 ± 0.8 min^-1^ and a catalytic efficiency of 0.5 ± 0.08 μM^-1^min^-1^. Remarkably, caspase-7 (0.4 μM) did not show any cleavage of this substrate (S7 and S8 Figs).

### Detecting caspase-3 activity in live cells

**Fig 4:**
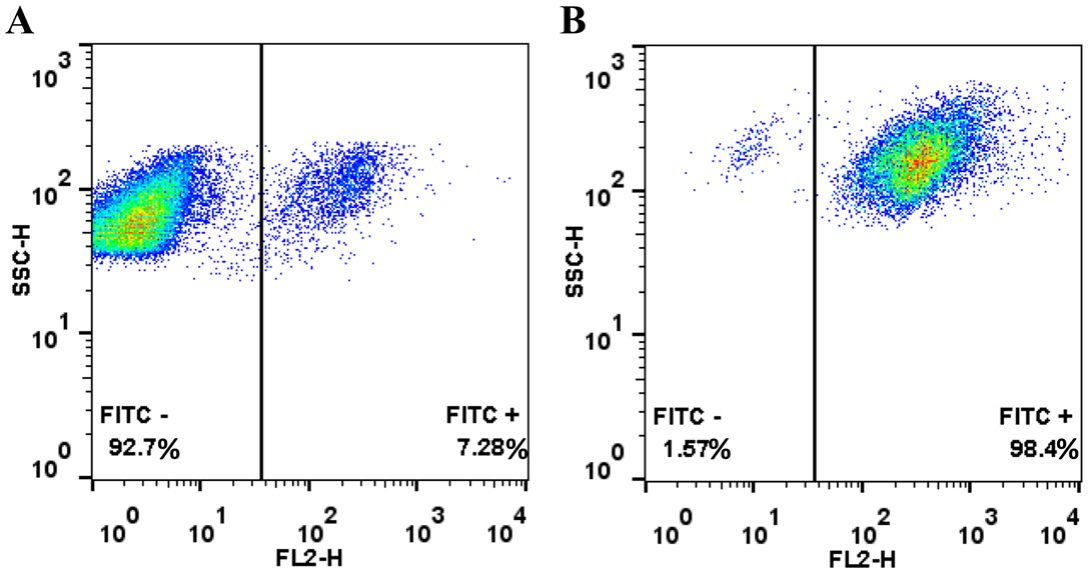
Flow cytometry analysis of healthy and apoptotic HEK293T cells treated with substrate 27. The cells were incubated 5 h with fluorogenic substrate **27** (10 μM), detached, and analysed by flow cytometry (λ_Ex/Em_ 488/530 nm, x-axis = fluorescence intensity). (A) Healthy, non-apoptotic cells. (B) Apoptotic cells (induced by 1 μM staurosporine).

**Fig 5:**
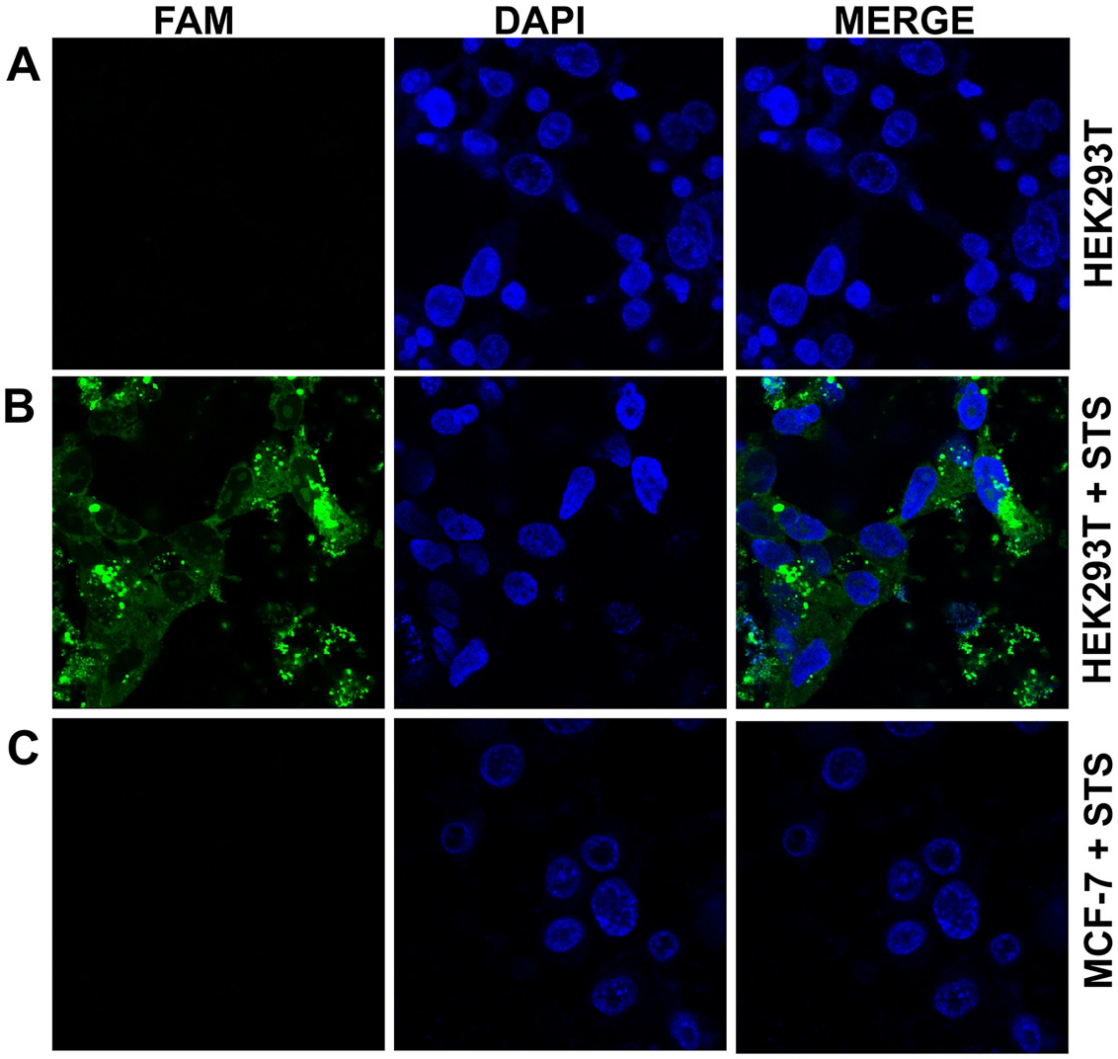
Confocal microscopy images of HEK293T and MCF-7 cells treated with substrate 27. Confocal microscopy images (objective HCX PL APO ×63/1.40–0.6 Oil CS) of HEK273T cells with substrate **27** (10 μM) without staurosporine (STS) (**A**) and with staurosporine (1 μM) (**B)** induced apoptosis (green fluorescence is from fluorescein “turned on” by caspase-3 cleavage of the substrate, blue is DAPI nuclear stain). (**C**) Staurosporine treated MCF-7 cells with substrate **27**.

Caspase-3 activity in HEK293T cells was evaluated using the caspase-3 selective substrate **27** with apoptosis induced by staurosporine. Flow cytometry analysis of cells treated with **27** (10 μM) showed a 2.5-fold increase in the fluorescence intensity (λ_Ex/Em_ 488/530 nm) of the cells after apoptotic stimulation by staurosporine (1 μM), with no increase observed in fluoresce without it (Fig 4). This increase in fluorescence with substrate **27** suggested that the concentration of caspase-3 in apoptotic cells was approximately 15.7 ± 0.5 nM per cell (28271 molecules per cell) in the execution phase of apoptosis (based on the *Vmax* and the *kcat* of **27**, see supporting information) [50]. In live-cell confocal imaging of caspase-3 activation with **27** (10 μM), fluorescence “turn-on” was only detected in the cytoplasm of apoptotic HEK293T cells, with no increase in fluorescence observed in non-apoptotic cells (Fig 5A, 5B and S9 Fig). No fluorescence “turn-on” was observed in apoptotic MCF-7 cells (Fig 5C), which lack functional caspase-3 but express caspase-7 [34,51,52], confirming the isoform selectivity. Substrate **27** was nontoxic in an MTT assay at 10 μM concentration (S10 Fig).

## Conclusions

Fluorogenic, FRET-based substrates for monitoring the enzymatic activity of caspase-3 *in situ* were synthesised with permutations of the substrate sequence Asp-X_2_-X_3_-Asp with the aim of improving selectivity for caspase-3 over caspase-7. The fluorogenic substrates had 5(6)-carboxyfluorescein in the amino-terminus and an optimised quencher, methyl red, introduced via Lys side chain modification. These fluorogenic substrates were conjugated at the C-terminus to a cationic cell delivery vehicle to allow efficient cellular uptake. Substrates with Pro in the X_2_ position (instead of Val), showed selectivity for caspase-3 over caspase-7, along with increased caspase-3 affinity, particularly when X_3_ was Ala, Leu or Val. Mass spectrometry studies revealed unexpected cleavage pattern of the fluorogenic substrates with caspase-7, while optimisation of the substrate via spacer relocation yielded a caspase-3 selective substrate **27** (FAM-Ahx-Asp-Leu-Pro-Asp-Lys(MR)-Ahx-peptoid). In apoptotic cells, the optimised substrate **27** allowed imaging of caspase-3 activity *in situ*. Flow cytometry analysis gave approximate quantification of the concentration of caspase-3 in a cell to be 16 nM (28271 molecules per cell). Future work is aimed at the use of this caspase-3 selective sequence in *in vivo* near-infrared fluorescence imaging techniques, especially for cancer, which require stable, highly specific, and sensitive fluorogenic substrates.

## Materials and Methods

### Synthesis of fluorogenic substrates 1–27

A highly optimised microwave-based solid–phase strategy was used to synthesise the cell penetrating peptide–peptoids [38]. All solvents and reagents were obtained from commercial suppliers and used without purification. A Rink-amide functionalised aminomethyl polystyrene (1% DVB, 100–200 mesh, loading 1.2 mmol/g) resin was used for the synthesis of the peptides with an Fmoc-based strategy (S1 Fig). **Coupling of the Fmoc-amino acids and fluorophores:** The resin (1 eq) was pre-swollen in DCM, washed with DMF, and added a pre-activated mixture (10 min) of the carboxylic acid (3 eq), DIC (3 eq) and Oxyma (3 eq) in DMF (0.1 M). This reaction mixture was stirred in the microwave (Biotage Initiator) for 20 minutes at 60°C after which the resin was washed with DMF, DCM and MeOH. **Fmoc deprotection:** The resin was shaken with 20% piperidine in DMF (2 ×10 min), and subsequently washed with DMF, DCM and MeOH. **Dde deprotection:** The resin was shaken with 2% hydrazine in DMF (v/v) (2 ×10 min), and subsequently washed with DMF, DCM and MeOH. **Cleavage from the resin and deprotection:** A solution of TFA/TIS/DCM (90:5:5) was added to the resin (20 μL of the cleavage cocktail per mg of resin) and left to shake for 5 hours. The resin was filtrated and washed with DCM, and the collected filtrate was evaporated under reduced pressure and the compound precipitated using cold diethyl ether. Peptides **1**–**24** were purified by preparative HPLC and analysed by MALDI-TOF MS. For the characterisation of the peptides, see S1 Table.

### Kinetic assays with caspase-3 and 7

Caspase-3 or caspase-7 (R&D systems, USA) was added to 100 μL of caspase assay buffer with substrates **1–14** at concentrations from 0.1 μM to 8 μM in a 96-well plate (n = 3) to give final enzyme concentration of 20 nM (**1**–**9**) or 15 nM (**10**–**14**). Fluorescence (λ_Ex/Em_ 485/528 nm) was recorded on a Biotek Synergy HT Multi-Mode Microplate Reader every 2 min. Control samples had the same composition but no enzyme. The rate (μM/min) was calibrated using a 5(6)-carboxyfluorescein conversion factor (0.0055 μM/RFU) and data plotted against time (min). For initial cleavage rate (0–5 min), plots were fitted using linear regression analysis and the Michaelis-Menten data generated using GraphPad Prism 5.

### Caspase-3 detection in live cells

Cell culture was performed in a Heracell 150 incubator (Heraeus) and in a Herasafe KS 18 class II negative-flow cabinet (Heraeus). HEK293T and MCF-7 cells (cultured in high glucose (4.5 mg/mL) DMEM supplemented with 4 mM glutamine, 100 units/mL penicillin, 10 mg/mL streptomycin and 25 mg/mL amphotericin B, and 10% FBS) were seeded onto a 48-well plate at a density of 10^4^ cells per well. After 12 hours, the media was removed and substrates **1**–**14** added at 10 μM in fresh media. Selected wells were also treated with staurosporine (1 μM). After 5 hours, the cells were washed twice with PBS, detached with trypsin/EDTA, harvested with 2% FBS in PBS supplemented with Trypan Blue (0.04%) for analysis on a BD FACSAria^®^ flow cytometer. Fluorescence was evaluated as mean fluorescence intensity (MFI) and estimated <100 u.a. for untreated control cells (consistent values independently of staurosporine addition). Apoptotic cells treated with substrate **1** were used as a positive control to obtain the maximum of fluorescence signal. 50,000 events per sample were plotted in two-dimensional dot plots based on forward and side scattering. The cellular size and complexity (SSC-H vs. FSC-H) were used to gate two populations (alive/apoptotic cells) (debri excluded). The data were analysed using the software Flowjo^®^ 7.5.

For confocal microscopy, the cells were fixed with 4% paraformaldehyde in PBS and the nuclei stained with Hoechst-33342 (1% w/v in PBS). Cellular fluorescence of cells was analysed using an Inverted Leica DM IRB with filter I3 (450–490 nm) and a Leica SP5 Confocal (FITC and DAPI channel).

## Supporting Information

S1 Fig Solid phase synthesis of the fluorogenic substrates 1–24. (PDF)

S2 Fig MALDI-TOF MS spectra of substrate 11. (PDF)

S3 Fig MALDI-TOF MS spectra of substrate 12. (PDF)

S4 Fig MALDI-TOF MS spectra of substrate 14. (PDF)

S5 Fig MALDI-TOF MS spectra of substrate 25. (PDF)

S6 Fig MALDI-TOF MS spectra of 26. (PDF)

S7 Fig Analysis of substrate 27 with caspase-3 and caspase-7. (PDF)

S8 Fig MALDI-TOF MS spectra of 27. (PDF)

S9 Fig Substrate 27 selectively labels apoptotic cells. (PDF)

S10 Fig Cell viability. (PDF)

S1 File Quantification of caspase-3 in apoptotic cells by flow cytometry. (PDF)

S1 Table MALDI-TOF MS and HPLC analysis of substrates 1–27. (PDF)
