# Supplementary material for: Fluorogenic Substrates for In Situ Monitoring of Caspase-3 Activity in Live Cells
Source: PLoS One. 2016 May 11;11(5):e0153209. doi: 10.1371/journal.pone.0153209 (PMC4864350; doi:10.1371/journal.pone.0153209)
Supplement: S2 Fig — (PDF) [file pone.0153209.s002.pdf]

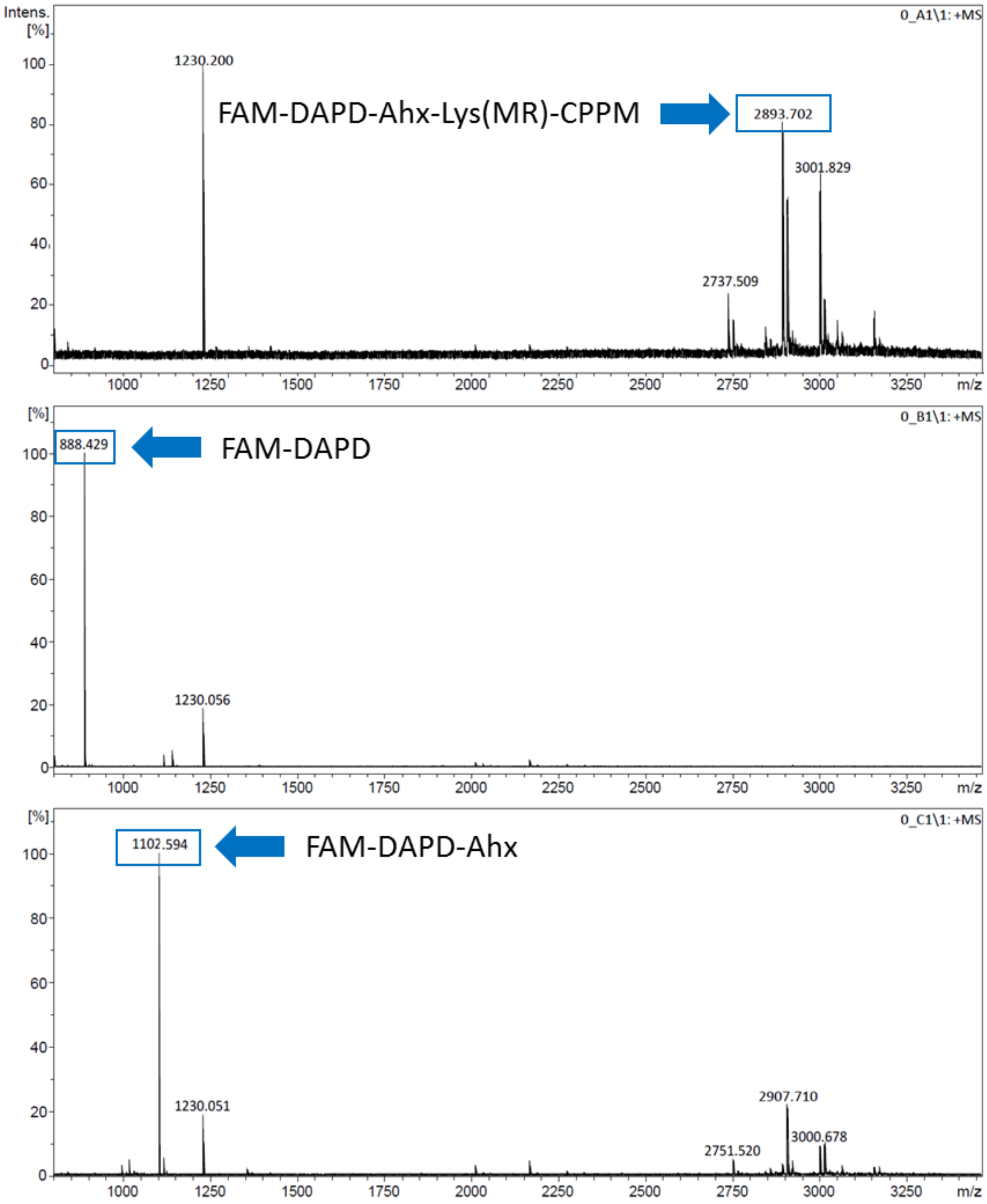

S2 Fig. (A) MALDI-TOF MS spectra of substrate **11** (sequence Asp-Ala-Pro-Asp). (B) After incubation (20 µM) with Caspase-3 for 2 h. (C) After incubation (20 µM) with Caspase-7 for 2 h (detected as the PBS adduct.)

Caspase-3 or caspase-7 (R&D systems, USA) was added to 100 µL of caspase assay buffer (final enzyme concentration 0.4 µM) with the substrates at 10–20 µM. The samples were incubated in Eppendorf Thermomixer® Comfort shaking at 1200 rpm for 2h at 37 °C. MALDI-TOF mass spectra were recorded as described in Table S1 .
