# Supplementary material for: Fluorogenic Substrates for In Situ Monitoring of Caspase-3 Activity in Live Cells
Source: PLoS One. 2016 May 11;11(5):e0153209. doi: 10.1371/journal.pone.0153209 (PMC4864350; doi:10.1371/journal.pone.0153209)
Supplement: S3 Fig — (PDF) [file pone.0153209.s003.pdf]

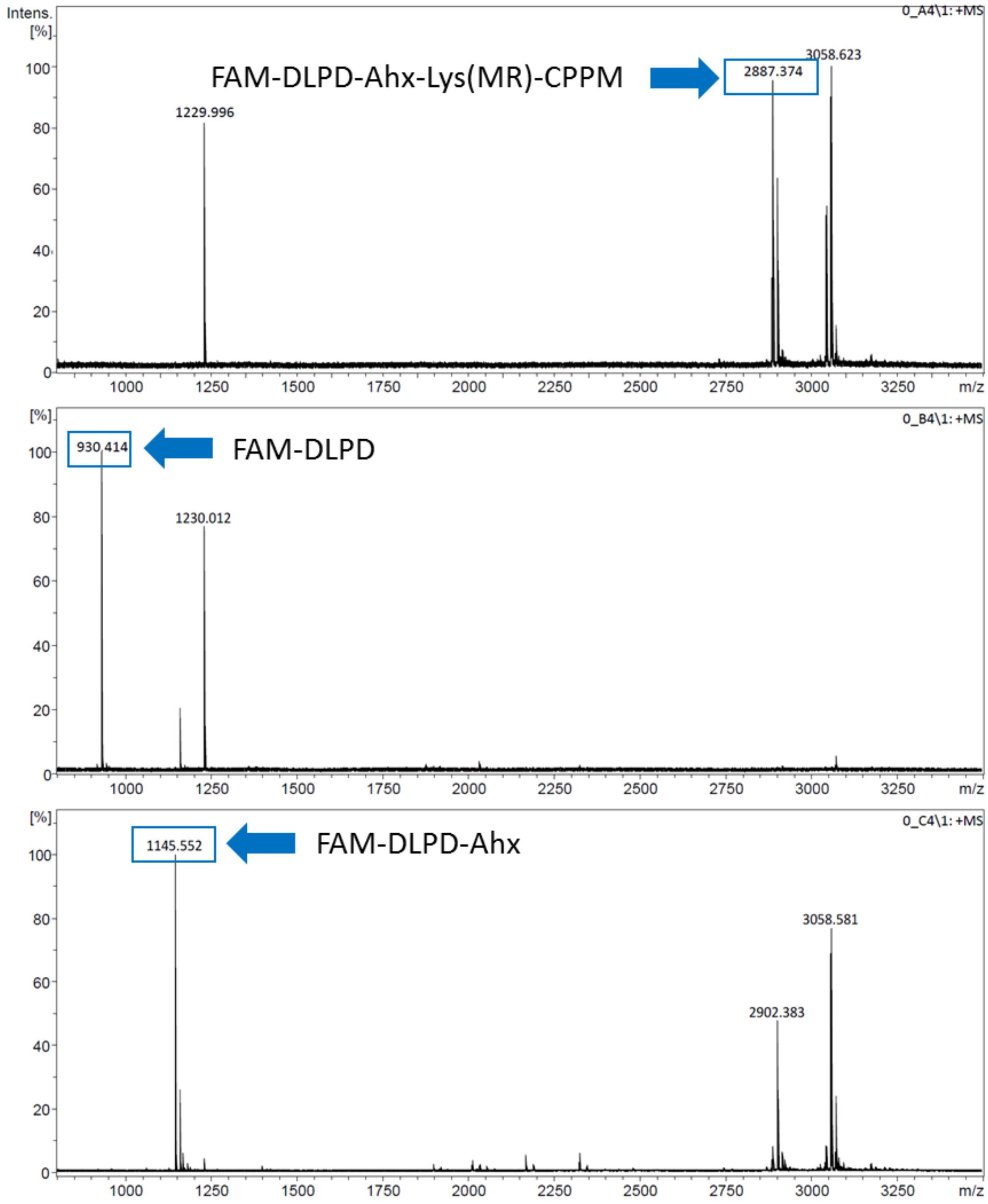

S3 Fig. A) MALDI-TOF MS spectra of substrate **12** (sequence Asp-Leu-Pro-Asp). (B) After incubation (20 μM) with Caspase-3 for 2 h. (C) After incubation (20 μM) with Caspase-7 for 2 h (detected as the PBS adduct.)
