# Supplementary material for: Fluorogenic Substrates for In Situ Monitoring of Caspase-3 Activity in Live Cells
Source: PLoS One. 2016 May 11;11(5):e0153209. doi: 10.1371/journal.pone.0153209 (PMC4864350; doi:10.1371/journal.pone.0153209)
Supplement: S4 Fig — (PDF) [file pone.0153209.s004.pdf]

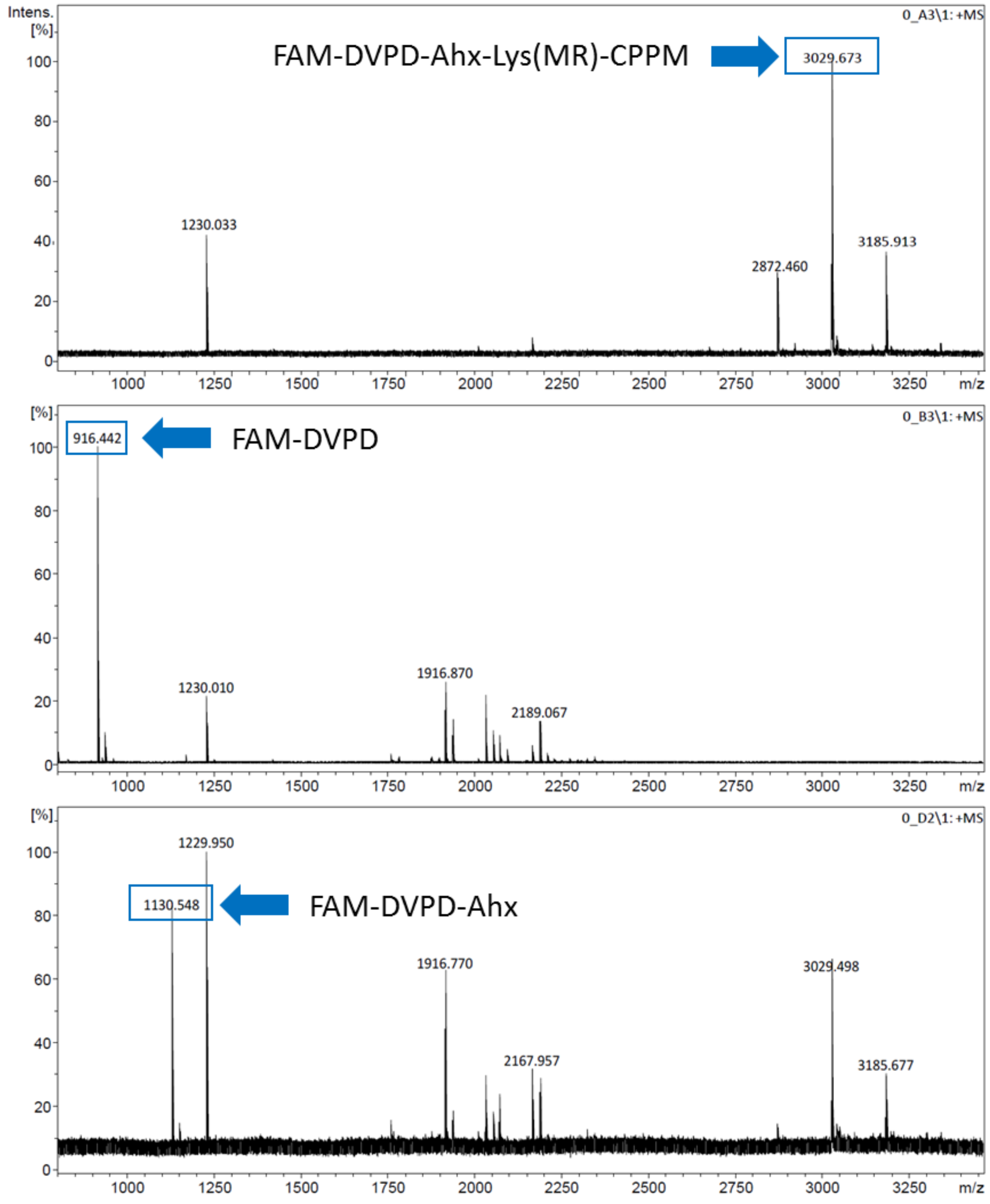

S4 Fig. A) MALDI-TOF MS spectra of substrate **14** (sequence Asp-Val-Pro-Asp). (B) After incubation (20 µM) with Caspase-3 for 2 h. (C) After incubation (20 µM) with Caspase-7 for 2 h (detected as the PBS adduct.)
