# Supplementary material for: Fluorogenic Substrates for In Situ Monitoring of Caspase-3 Activity in Live Cells
Source: PLoS One. 2016 May 11;11(5):e0153209. doi: 10.1371/journal.pone.0153209 (PMC4864350; doi:10.1371/journal.pone.0153209)
Supplement: S7 Fig — (PDF) [file pone.0153209.s007.pdf]

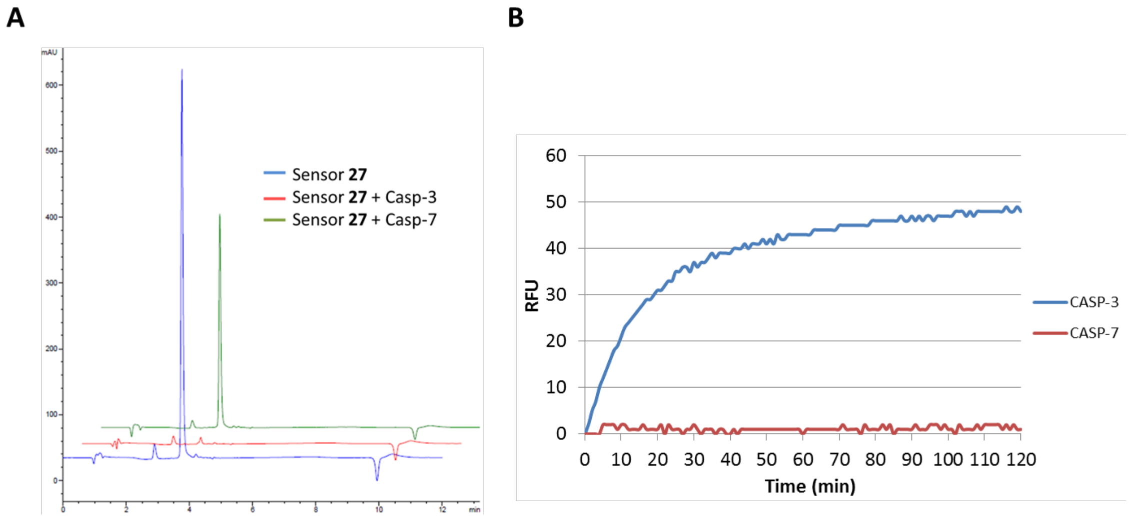

S7 Fig. (A) HPLC chromatogram (detection at 254 nm) of substrate **27** (10 μM) after 120 min incubation with caspase-3 or caspase-7 (0.3 μM). (B) Time-dependent increase in fluorescence ($\lambda_{Ex/Em}$ 485/528 nm) of **27** (0.6 μM) during 120 min incubation with caspase-3 or -7 (15 nM).
