# Supplementary material for: Fluorogenic Substrates for In Situ Monitoring of Caspase-3 Activity in Live Cells
Source: PLoS One. 2016 May 11;11(5):e0153209. doi: 10.1371/journal.pone.0153209 (PMC4864350; doi:10.1371/journal.pone.0153209)
Supplement: S8 Fig — (PDF) [file pone.0153209.s008.pdf]

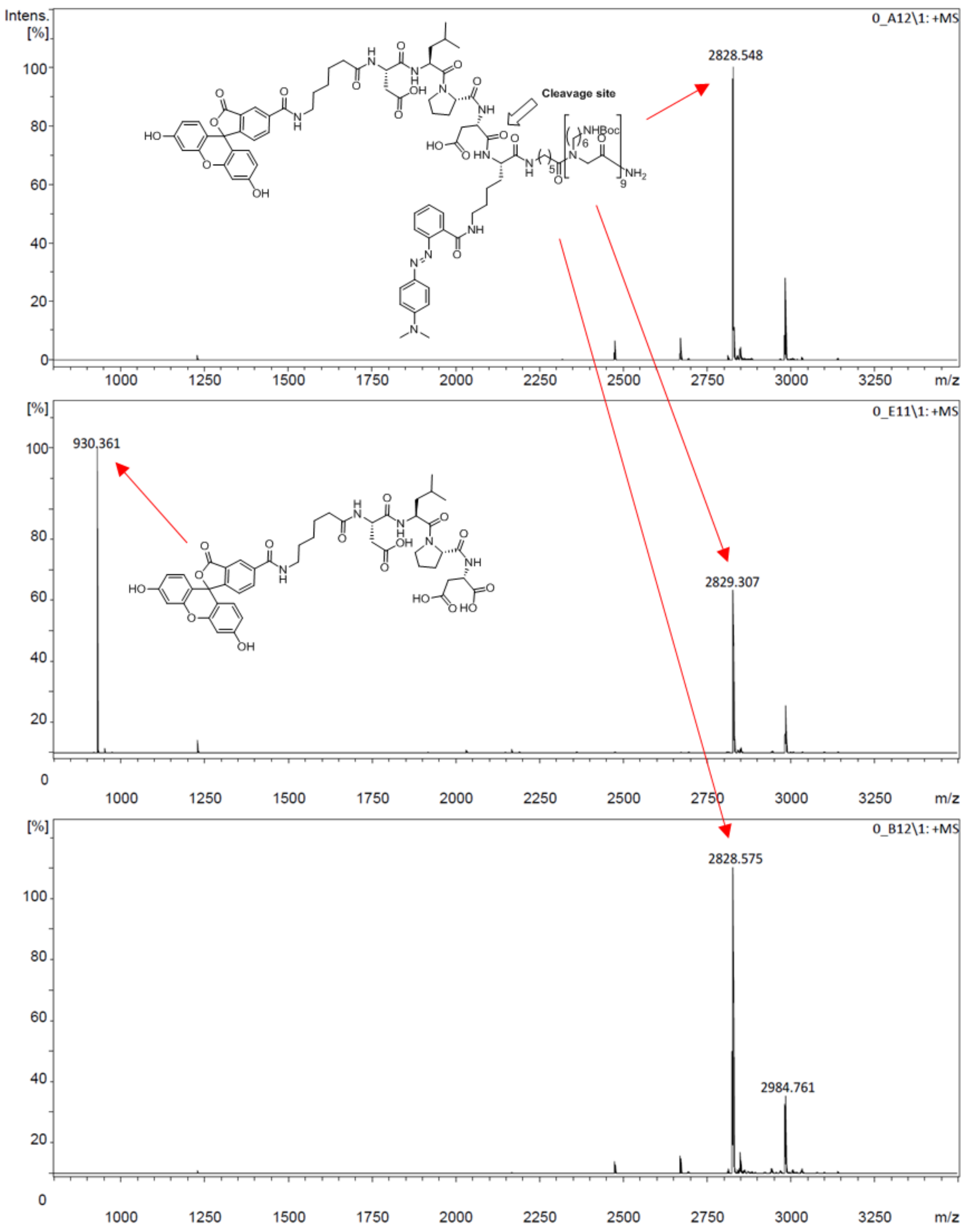

S8 Fig. MALDI-TOF MS spectra of **27** (10 µM) (**top spectra**), **27** (10 µM) incubated with caspase-3 (0.3 µM) for 2h (**middle spectra**), and **27** (10 µM) incubated with caspase-7 (0.3 µM) for 2h (**bottom spectra**).
