# Supplementary material for: Fluorogenic Substrates for In Situ Monitoring of Caspase-3 Activity in Live Cells
Source: PLoS One. 2016 May 11;11(5):e0153209. doi: 10.1371/journal.pone.0153209 (PMC4864350; doi:10.1371/journal.pone.0153209)
Supplement: S9 Fig — (PDF) [file pone.0153209.s009.pdf]

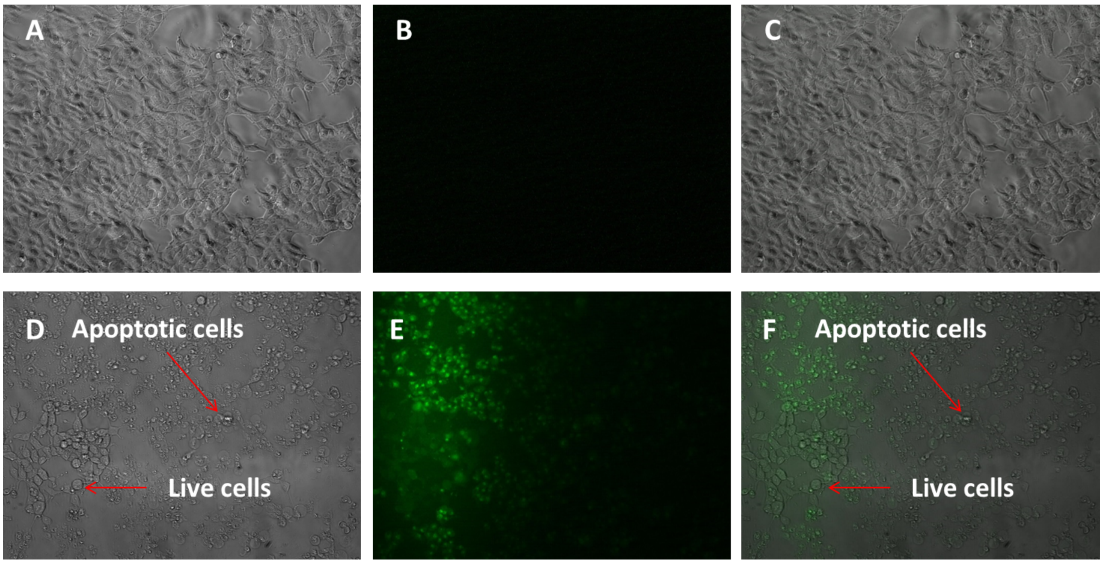

S9 Fig. Top: Microscope images (A = brightfield, B = Fluorescein, C = merge) of healthy HEK293T cells incubated with substrate **27 (**10 μM). Bottom: HEK293T cells incubated with staurosporine (1μM) and substrate **27** (10 μM)(D = brightfield, E = Fluorescein, F = merge) with only apoptotic cells exhibiting fluorescence.
