# Supplementary material for: Fluorogenic Substrates for In Situ Monitoring of Caspase-3 Activity in Live Cells
Source: PLoS One. 2016 May 11;11(5):e0153209. doi: 10.1371/journal.pone.0153209 (PMC4864350; doi:10.1371/journal.pone.0153209)
Supplement: S10 Fig — (PDF) [file pone.0153209.s010.pdf]

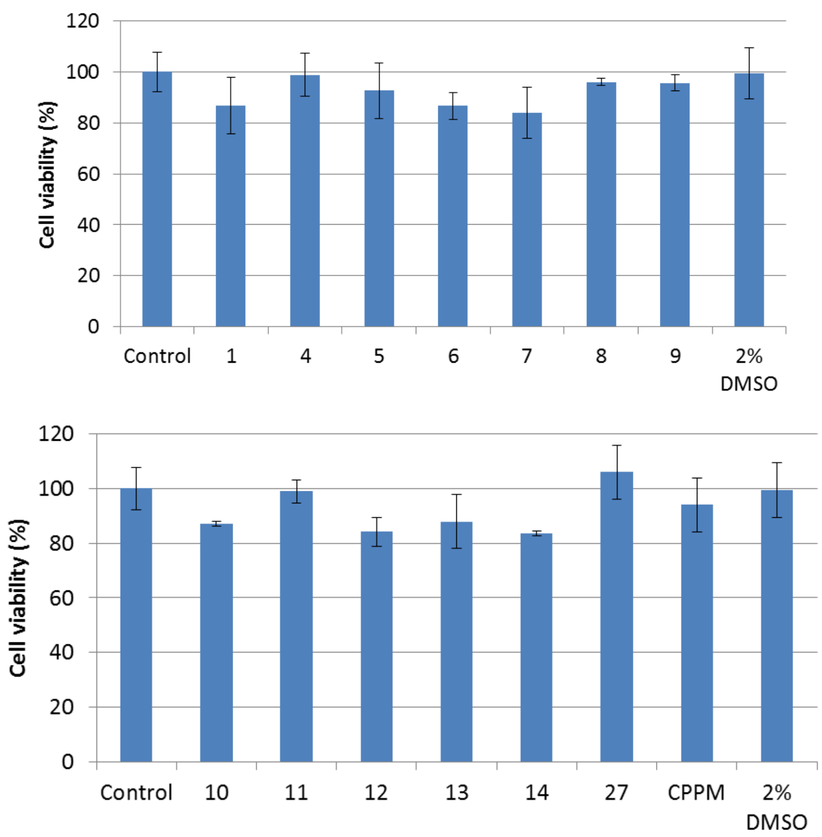

S10 Fig. Cell viability (MTT) assay of HEK293T cells after incubation with the fluorogenic substrates at 10 µM (n = 8). Cell viability was recorded using a BioTek microplate reader Synergy HT measuring absorbance at 490 and 630 nm.
