# Supplementary material for: Fluorogenic Substrates for In Situ Monitoring of Caspase-3 Activity in Live Cells
Source: PLoS One. 2016 May 11;11(5):e0153209. doi: 10.1371/journal.pone.0153209 (PMC4864350; doi:10.1371/journal.pone.0153209)
Supplement: S1 Fig — (PDF) [file pone.0153209.s001.pdf]

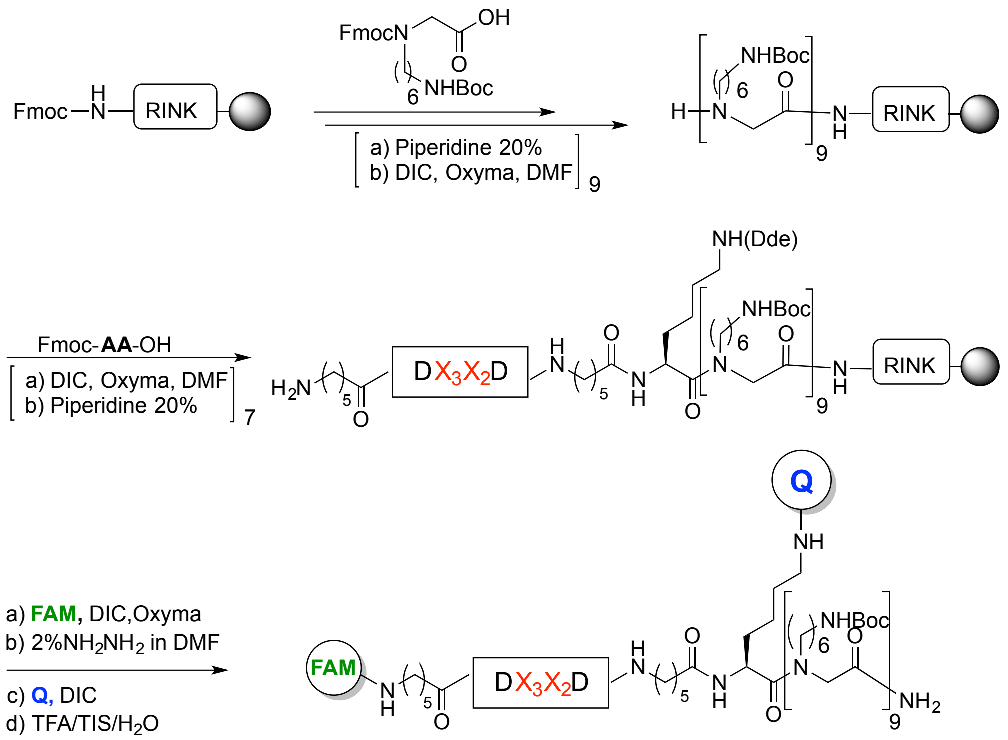

1–24

S1 Fig. Solid phase synthesis of the fluorogenic substrates **1–24**.
