# Supplementary material for: Fluorogenic Substrates for In Situ Monitoring of Caspase-3 Activity in Live Cells
Source: PLoS One. 2016 May 11;11(5):e0153209. doi: 10.1371/journal.pone.0153209 (PMC4864350; doi:10.1371/journal.pone.0153209)
Supplement: S1 Table — (PDF) [file pone.0153209.s012.pdf]

S1 Table. MALDI-TOF MS and HPLC analysis of substrates 1–27. MALDI-TOF mass spectra were performed using a Bruker UltraflexExtreme MALDI TOF/TOF instrument and analysed with the Bruker Daltonics flexAnalysis software. For peptide/peptoid analysis, sinapic acid was used as a matrix and positive ion mass spectra were recorded. HPLC analyses were performed on an Agilent 1100 analytical system equipped a Supelco Discovery® C18 column (5 µm, 5 cm) coupled to a Polymer laboratories 100 ES evaporative light scattering detector (ELSD). Eluting from 5% A (H_2O + 0.1% formic acid) to 95% (MeOH + 0.1% formic acid) over 8 min, followed by isocratic 95% B for 1 min, at a flow rate of 1 mL/min. Semipreparative HPLC purifications were performed on an Eclipse XDB-C18 column (9.4 x 250 mm, 5 µm), at a flow rate of 2 mL/min, with detection/collection at 254 nm. The following gradient was used: 5 to 50 % A over 6 min, 50 to 100% B for 30 min.

	**Sequence**	**MW [calc.]**	**MALDI-TOF**	**HPLC purity (%)**
**1**	DEVD	$(M+H)^+$ 2845.8	2845.1	100
**2**	DEVD	$(M+H)^+$ 2837.7	2837.1	92
**3**	DEVD	$(M+H)^+$ 2883.7	2883.9	75
**4**	DPVD	$(M+H)^+$ 2813.8	2813.3	93
**5**	DGVD	$(M+H)^+$ 2774.6	2774.2	97
**6**	DAVD	$(M+H)^+$ 2788.6	2788.3	99
**7**	DLVD	$(M+H)^+$ 2829.8	2829.4	96
**8**	DNVD	$(M+H)^+$ 2829.8	2829.4	98
**9**	DVVD	$(M+H)^+$ 2816.6	2816.3	99
**10**	DGPD	$(M+TFA-H)^+$ 2881.7	2881.6	93
**11**	DAPD	$(M+TFA-H)^+$ 2895.7	2895.5	95
**12**	DLPD	$(M+TFA-H)^+$ 2937.8	2934.3	90
**13**	DNPD	$(M+TFA-H)^+$ 2938.7	2937.4	94
**14**	DVPD	$(M+TFA-H)^+$ 2923.7	2923.0	100
**15**	dgvd	$(M+Na+K)^+$ 2832.7	2832.4	100
**16**	davd	$(M+Na+K)^+$ 2847.7	2847.5	100
**17**	dlvd	$(M+Na+K)^+$ 2889.7	2889.3	100
**18**	dnvd	$(M+Na+K)^+$ 2890.7	2890.3	100
**19**	dvvd	$(M+Na+K)^+$ 2875.7	2875.4	100
**20**	dgpd	$(M+Na+K)^+$ 2831.7	2831.4	100
**21**	dapd	$(M+Na+K)^+$ 2845.7	2845.5	100
**22**	dlpd	$(M+Na+K)^+$ 2887.7	2887.4	100
**23**	dnpd	$(M+Na+K)^+$ 2888.7	2888.3	100
**24**	dvpd	$(M+Na+K)^+$ 2873.7	2873.4	100
**25**	DLPD-Ahx-k	$(M + H)^+$ 2827.7	2827.5	100
**26**	DLPD-Ahx-K(Me)	$(M + 2Na - H)^+$ 2883.6	2883.9	100
**27**	DLPD-K-Ahx	$(M + H)^+$ 2827.7	2827.7	100
