# Supplementary material for: Fluorogenic Substrates for In Situ Monitoring of Caspase-3 Activity in Live Cells
Source: PLoS One. 2016 May 11;11(5):e0153209. doi: 10.1371/journal.pone.0153209 (PMC4864350; doi:10.1371/journal.pone.0153209)
Supplement: S1 File — (PDF) [file pone.0153209.s011.pdf]

Quantification of caspase-3 in apoptotic cells by flow cytometry

Localisation of apoptotic cells by flow cytometry. Flow cytometry analysis of morphology (SSC-A) and size (FSC-A) of HEK293T cells without (left image) and with (right image) treatment with 1 µM staurosporine for 4 h.

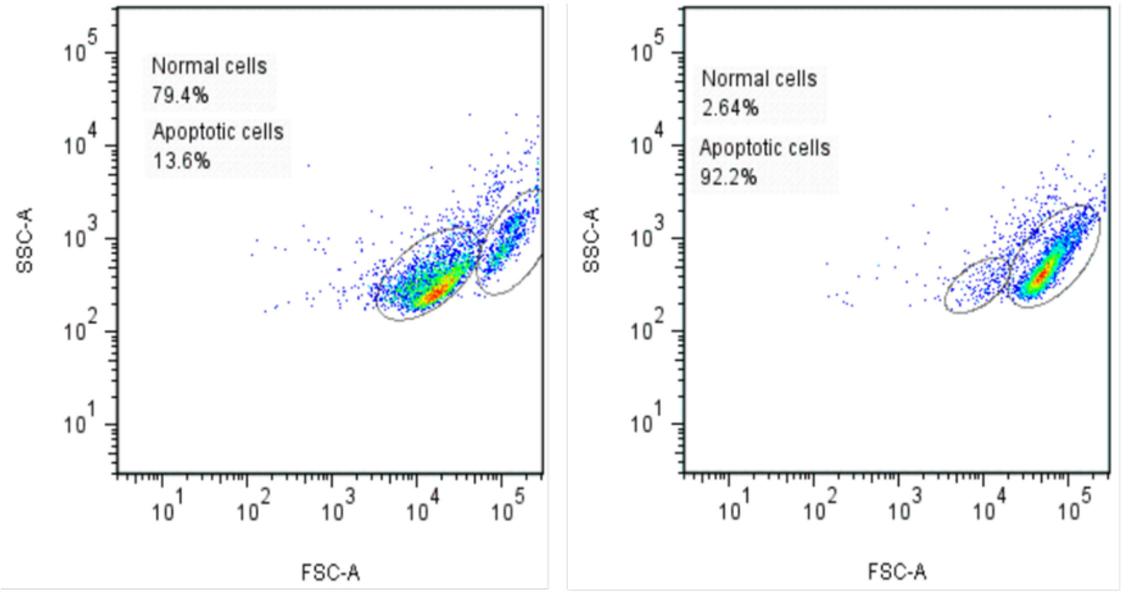

Calculation of active caspase-3 concentration inside cells

Substrate **27** was used to quantify caspase-3 per cell.[1]

1. Calculate FAM-Ahx-Leu-Pro-Asp released per cell:

Fluorescein-labelled microspheres (0.5% SC, ≈ 2 mmol/g),[2] were used to generate the standard curve for fluorescence intensity versus amount of the fluorophore (nmol) by flow cytometry (see below).

[1] Kossler, S.; Nofziger, C.; Jakab, M.; Dossena, S.; Paulmichl, M. *Toxicology*, **2012**, *292*, 123-135.
[2] a) A. uncit-Broceta, E.M.V. Johansson, R.M. Yusop, R.M. Sanchez-Martin and M. Bradley, *Nat. Protocol*, **2012**, *7*, 1207-1218. b) J.M. Cardenas-Maestre and R.M. Sanchez-Martin, *Org. Biomol. Chem.*, **2011**, *9*, 1720-1722.

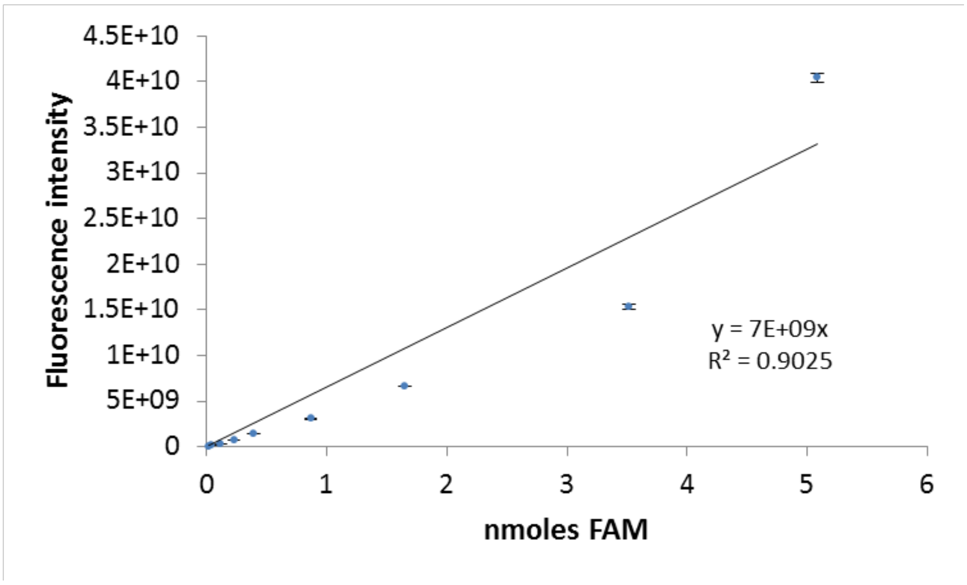

FAM standard curve to determine conversion factor (7x10^9 MFI/nmol) representing FI (Fluorescence Intensity) versus FAM quantity (nmol).

To determine the concentration of FAM-Ahx-Leu-Pro-Asp released per cell (nmol/cell) = [FI/cell – FI_0/cell]/7x10^9 FI/nmol

Assuming 20 µm cell diameter and 2/3 of that for the nucleus, the cytoplasmic volume of HEK293T cell is 3 pl (VC).[1,3]

FAM-Ahx-Leu-Pro-Asp released per cell (M) = FAM-Ahx-Leu-Pro-Asp released per cell (fmol/cell)/3000 fl

2. Calculate the rate of product formation V_0 with 5 h staurosporine treatment:

V_0 = d[P]/dt = FAM-Ahx-Leu-Pro-Asp released per cell (mM)/18000 sec

3. Quantify the concentration of the enzyme per cell at high substrate concentrations (enzyme saturation)

kcat = *Vmax*/[E]

kcat = 0.04 s^{-1} (sensor **27** DLPDK)

[E] = enzyme concentration per cell (nM)

[Caspase-3] = 15.65 ± 0.46 nM (1.3 ng active caspase 3 per 10^6 apoptotic staurosporine-treated HEK293T cells).[4]

[3] Y. Liu, X. Yan and T. Zhou, *PLOS one*, **2013**, *8,* 8.

4. Quantify the number of molecules per cell of caspase-3

N^{o}E (number of enzyme molecules per cell) = [E]xVCxNA

NA = 6.023 x 10^{23} molecules/mol

N^o Cas3 = 28271.22 molecules/cell

Sensor 27	fmol FAM/cell	FAM (mM)	V (mM)/min	[E] (nM)	E molecules/cell
**Average**	0.029571429	0.00766667	0.009857143	15.65	28271.22
**SD ±**	0.000868966	0.00022529	0.000289655	0.46	830.76

[4] Roff, C. F. *et al*, *Anal. Biochem*. **2000**, *284*, 114-124.
